# Long-Term Water Balance Evaluation in Glass Ionomer Restorative Materials

**DOI:** 10.3390/ma15030807

**Published:** 2022-01-21

**Authors:** Howard Roberts, David Berzins, John Nicholson

**Affiliations:** 1 Dental Biomaterials Research, College of Dentistry, University of Kentucky, Lexington, KY 40536, USA; 2 Graduate Dental Biomaterials, School of Dentistry, Marquette University, Milwaukee, WI 53233, USA; david.berzins@marquette.edu; 3 Bluefield Centre for Biomaterials, UK and Dental Physical Sciences, Barts & The London School of Medicine and Dentistry, Queen Mary University of London, London E1 4NS, UK; john.nicholson@bluefieldcentre.co.uk

**Keywords:** glass ionomer, polyalkenoate, water content, unbound water, bound water

## Abstract

The complex role of water in glass ionomer cement (polyalkenoate) dental restorative materials has been studied, but much of the present understanding concerning water balance within these materials is based on very early studies and short-term experiments. This study evaluated the nature of the water species of six conventional and four resin modified glass ionomer restorative materials over 3 years using thermogravimetric analysis techniques. Materials were prepared, placed in crucibles, and stored in physiologic phosphate buffered saline and evaluated at 24 h, 1 week, and then at 1, 3, 6, 9, 12, 18, 24, 30 and 36 months. All materials demonstrated a significant increase in unbound water percentage content but except for the resin modified materials, the enthalpy required to remove the unbound water species did not significantly change over 36 months. Also, bound water content percentage and removal enthalpy was established at 24 h, as no significant increase was noted in both bound water content and removal enthalpy over the course of this evaluation. This study suggests that unbound water species may increase with time and is loosely held except for the resin modified materials. Protective coatings placement and re-evaluation are prudent to prevent unbound water loss.

## 1. Introduction

Glass ionomer cement (GIC) restorative materials remain a useful restorative dentistry option, allowing many different applications in clinical dentistry. GIC restorative materials have a proven clinical performance in the austere conditions of the Atraumatic Dental Technique [1,2,3]. Furthermore, conventional GIC restorative materials current formulations have been recently suggested in clinical evaluations under certain conditions to be an acceptable restoration material for posterior restorations subjected to functional forces [4,5,6].

Since the initial formulation of GIC materials by Wilson and Kent [7,8], GIC restorative materials have undergone many modifications to improve mechanical properties and clinical performance [9,10,11,12,13,14,15]. The setting process of GIC materials involves an acid-base reaction between a polyalkenoate and tartaric acid with an fluoroaluminosilicate glass [9]. The acid-base reaction is essentially complete by 24 h [9] but continued maturation is thought to consist of continued cross linking between polymer chains with a hydrogel matrix maturation, which corresponds with improvements in mechanical properties [16,17,18]. Resin-modified GICs (RMGICs) contain resin monomers that are added at the expense of water content which the monomer polymerizes by either a visible light curing (VLC) photoactivation or an internal chemical reaction [19,20], and research continues for optimal RMGI resin components [21]. The polyalkenoate acid-base reaction within RMGI materials is hindered by the resin content [22] with the initial resin matrix gelation inhibiting polyalkenoate diffusion [23,24], stereochemical distortion of the polyalkenoate acid chains [24], and reduced water content [25,26,27]. While 90 percent of resin polymerization is thought to be complete after photoactivation, some continued resin polymerization has been suggested, similar to that of resin composite materials [28]. However, it has been suggested that any post-photopolymerization resin reaction may be likewise hindered by the polyakeonate rection, as several reports suggest that both RMGIC resin monomer polymerization and polyalkenoate acid-base reaction both compete with and exert inhibitory effects on each other [25,26,27].

The setting reaction of GIC materials has been a subject of interest [29,30,31,32,33]. More specifically, the complex role of water in glass ionomer materials has also been a topic of research [34,35,36,37]. Importantly, water is required for the polyalkenoate reaction to occur—water is the medium in which the setting reaction occurs and is a component of the set cement—which is a frequently overlooked aspect [9]. Essentially all the water initially incorporated in the cement becomes a part of the set cement—there is no active water expulsion as the cement sets [9] and this water content maintenance not only determines the ultimate mechanical properties [9], but also mediates the setting processes [38] as well as contributes to the esthetics of the set material [39]. The need to prevent damage from excess water exposure and deter subsequent water loss is a well-known requirement [40,41,42,43,44].

Distinct water species are said to exist within glass ionomer materials: one species that was easily lost from the material (e.g., “loosely bound”) and another separate category that is more difficult to remove (e.g., “tightly bound”) [45,46]. In addition, a third but small superficial water species that does not require diffusion through the glass ionomer material has also been suggested [37]. Loosely bound water has been considered to be removed by exposure to a desiccant or to 105 °C heat for 24 h; while the tightly bound water defined as being more resistant to removal [45,46]. While arbitrary, these conditions do identify that water exists within different internal GIC locales, while also possessing a degree of diffusion mobility [47], albeit slow [36], with the internal distribution location possibly differing with time [9,47]. Wilson and Crisp estimated with initial GIC materials that 18–28% of the total glass ionomer water content was loosely bound while tightly bound water was thought to be approximately 5% [45]. More recent studies do somewhat collaborate the estimates of tightly bound water [37]. With increasing maturity, early studies suggested that the ratio of tightly bound to loosely bound water increases [46], and various mechanisms have been proposed to explain the mechanism. Some suggest the formation of strong hydrated ions from the Na^+^, Ca^+2^, Sr^+2^, and Al^+3^ cations released from the glass [36], the formation of a stable hydration sheath around the ionized polyacrylic polymer [21], while others credit formation of strongly-bonded silanol groups due to water interaction with the glass surface siloxane groups [9,48,49,50].

Although more recent studies elucidate some aspects of glass ionomer materials, much of present understanding concerning water balance within GIC materials is based on very early studies on these materials. Understandably, it is not known if modern-day glass ionomer materials possess the water dynamics and characteristics. The purpose of this study was gain information on the water species of six conventional and four resin modified glass ionomer (RMGI) restorative materials using thermal analysis techniques. The null hypothesis was that would be no difference in the water characteristics with and between the different materials over an extended time.

## 2. Materials and Methods

The materials evaluated in this study are listed in Table 1.

Materials were chosen based on prominence as identified in the scientific literature. Four not previously mentioned products were added as well. All materials were in encapsulated delivery systems, prepared following the manufacturer’s instructions, and injected directly into 40 μL aluminum differential scanning calorimetry (DSC) crucibles (Mettler-Toledo LLC, Columbus, OH, USA). Conventional GIC materials were allowed to set undisturbed without water exposure for the recommended setting time while RMGI products were photopolymerized for manufacturer recommended duration with a light emitting diode (LED) visible light curing (VLC) unit (Bluephase G2, Ivoclar Vivadent US, Amherst, NY, USA) of which irradiance output (~1100 mW/cm^2^) was verified with a radiometer (Bluephase Meter II, Ivoclar Vivadent US). Materials prepared in their respective crucibles were then weighed to the nearest hundredth of a milligram with an analytical digital balance (MS105, Mettler-Toledo).

**Figure 1:**
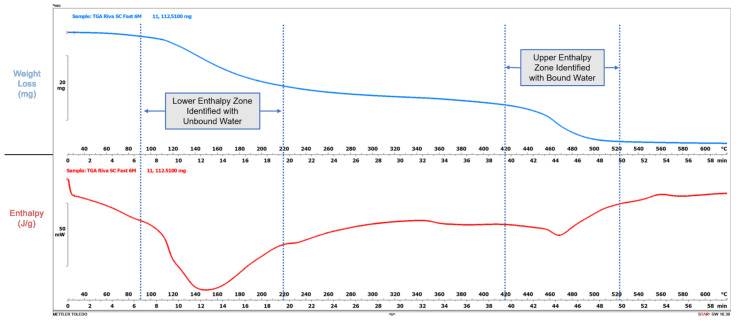
Thermal Analysis Protocol with Enthalpy Zones Identified.

This evaluation sought to evaluate moisture content stability in glass ionomer materials, so to establish optimal conditions specimens did not receive protective coatings to allow ample access to moisture. Samples (*n* = 12) were stored in a 95% 0.2 M phosphate buffered saline (PBS) environment as suggested by ISO 9917 [51]. To lessen chances of material loss due to solubility, specimen total immersion avoidance was attempted by placing the crucibles directly into a container in which the bottom surface was covered with several layers of PBS-saturated cotton gauze. The samples were then covered with several layers of dry cotton whose periphery was placed in contact with the PBS-saturated bottom layer. Thus, the glass-ionomer samples were supplied and maintained moisture through wicking action of the top layer of gauze. After moisture supply was observed the containers were sealed and stored in an incubator at 37 °C until time of testing. Additional PBS was occasionally added to the bottom gauze layer to ensure that the upper gauze layer covering the samples maintained a moist appearance. Testing occurred at 24 h, one week, and then at one, three, six, nine, 12, 18, 24, 30, and 36 months. At evaluation, specimens were weighed and then placed into a combined thermogravimetric/differential scanning calorimeter (TGA/DSC1, Mettler-Toledo). The thermogravimetric function recorded any weight change during the thermal challenge while the differential scanning calorimeter measured the enthalpy changes required to cause any weight change. Samples were subjected to a 25–620 °C thermal challenge at a rate of 10 °C per minute under a nitrogen purge atmosphere. Enthalpy curves and the associated weight loss were integrated at observed temperature ranges with enthalpy curves being integrated using a spline baseline method while weight loss was determined using a step-integrated method over the enthalpy curve range. Since sample sizes could not be standardized, all weight changes were derived as a percentage of the initial sample weight. As depicted in Figure 1, assumptions made for this evaluation were that essentially the lower temperature enthalpy curves and weight loss were associated with unbound water content. Additionally, the higher temperature enthalpy and weight loss were due to mainly bound water weight loss.

Admittedly these assumptions could be further refined with analysis of evolved gases with either mass spectroscopy or infrared analysis, however these modalities were not available during this evaluation. The following parameters were evaluated based on weight loss that was presumed to be largely water:

(1) Unbound water content and enthalpy, based on lower temperature enthalpy and weight loss; and
(2) Bound water content and enthalpy, based on higher temperature enthalpy and weight loss.

Mean results were subjected to Shapiro-Wilk and Bartlett’s Tests which identified irregularities in both the data distribution and variance homogeneity, respectively. Therefore, data was evaluated using Kruskal-Wallis with Dunn’s post hoc testing when required at a 95% level of confidence (α = 0.05).

## 3. Results

The mean results of the unbound water content loss are presented in Table 2.

The GIC materials all demonstrated a significant increase in unbound water percentage content over the evaluation period. Compared to that at 24 h, unbound water in Chemfil Rock significantly increased at 12 months (*p* = 0.001), then remained similar for the remaining time up to 36 months (*p* > 0.9999). Equia demonstrated a significant increase at 9 months (*p* < 0.0001) that was also maintained at 36 months (*p* > 0.9999). Ketac Fil demonstrated a significant unbound water increase at 6 months (*p* = 0.034) and despite some variation, maintained similar unbound water content as that at 36 months (*p* > 0.9999). Ketac Molar Quick required only 3 months to demonstrate a significant unbound water content (*p* = 0.023) that was stable until the end of the evaluation (*p* > 0.9999). Both Riva GIC products were noted to significantly increase unbound water content at 3 months (Riva SC Fast, *p* = 0.0035; Riva SC HV, *p* = 0.0027) which both products maintained until 36 months (Riva SC Fast, *p* > 0.9999; Riva SC HV, *p* = 0.27). The RMGI products also demonstrated significant increases at various times with Fuji II LC requiring 6 months to demonstrate a significant increase compared to 24 h (*p* = 0.017) but reached similarity with 36 months as early as 3 months (*p* = 0.082). Riva LC was similar to 36 month values as early as 1 week (*p* > 0.9999) while Riva LC HV demonstrated significant unbound water content at 6 months compared to 24 h (*p* = 0.0055) but reached similarity with 36 month content at 3 months (*p* > 0.9999). When the unbound water content was compared between materials, at 24 h all 3 RMGI materials had significant less unbound water content than Chemfil Rock, Equia, Ketac Fil, Ketac Molar Quick, and Riva SC Fast (*p* < 0.0078). With time, all RMGI materials reached unbound water content overall similarity with the GIC materials as soon as 1 month for Riva LC and Riva LC HV (*p* > 0.23) for that was uniformly reached at by all RMGI products by 18 months (*p* > 0.18).

The enthalpies involved with the unbound water content are presented in Table 3.

Chemfil Rock and Equia did not demonstrate any change in enthalpy required to remove unbound water content over 36 months (*p* = 0.85 and *p* = 0.078, respectively). Furthermore, Ketac Fil and Riva SC HV also did have an enthalpy requirement change required to remove the unbound water content (*p* = 0.071, *p* = 0.52, respectively). Ketac Molar Quick demonstrated a significantly lesser enthalpy requirement to remove unbound water at 6 and 9 months (*p* > 0.011), but otherwise all other testing time results were similar (*p* > 0.9999). Riva SC Fast was observed to have no enthalpy requirement differences between 24 h and 36 months (*p* > 0.061) except for reduced enthalpy requirements at 1 month (*p* = 0.048), 3 months (*p* = 0.0009). In regards to the RMGI materials, Fuji II LC at 24 h demonstrated the lowest numerical enthalpy required to remove unbound water that continued similarity up to 6 months (*p* = 0.48) but achieved significant greater values as compared to 24 h at 9 months (*p* = 0.0014). However, similarity to 36 month enthalpy values was found as early as 3 months (*p* > 0.9999). Riva LC established similar enthalpy seen at 36 months at 1 week (*p* > 0.9999) while Riva LC HV required 30 months to establish significantly greater enthalpy as seen at 24 h (*p* = 0.0039). However, Riva LC HV enthalpy values increased to achieve similarity to 36 months values at 1 month (*p* = 0.33). When products were compared at the different testing intervals are reviewed, at 24 h all GIC restorative materials demonstrated significantly greater unbound water enthalpy values as compared to the RMGI materials (*p* < 0.0097). At one week Riva LC enthalpy increased to be similar with the GIC products (*p* = 0.019), while at 1 month both Riva GIC products demonstrated lower unbound water enthalpy that was similar to both Riva LC and Riva LC HV (*p* > 0.9999). At 3 months all RMGI products were similar to the GIC materials (*p* > 0.9999) which was essentially maintained for the remainder of the evaluation.

The mean bound water content as a percentage of sample total weight is shown in Table 4.

When considered against the total sample weight there were no significant differences in bound water content noted within Chemfil Rock (*p* = 0.065), Equia (*p* = 0.054), Ketac Molar Quick (*p* = 0.11), Riva LC (*p* = 0.055), Riva LC HV (*p* = 0.061), Riva SC Fast (*p* = 0.078) and Riva SC HV (*p* = 0.068). Fuji II LC had significantly greater % bound water content starting at 12 months as compared to that of one, three, and 6 months (*p* < 0.024) but considerable similarity overlap was noted. Ketac Fil was noted to demonstrate significantly greater % bound water content up to nine months as compared to 18 months (*p* = 0.0065). Ketac Fil bound water loss was in an overall declining trend after nine months but maintained similarity to nine-month values at 36 months (*p* = 0.096). When the percentage bound water content is evaluation between the materials at noted evaluation times, significant differences are identified but most material results existed within a wide similarity overlap. Overall, Ketac Molar Quick consistently was noted as demonstrating significantly less bound water content (*p* < 0.036) at all time intervals. Fuji II LC was also identified from 1 to 6 months as demonstrating significantly less bound water content (*p* < 0.037) but after 6 months was similar to the other materials.

The mean enthalpy results associated with the bound water content is shown in Table 5.

When bound water enthalpy is considered, Table 5 shows that there were no significant differences identified over the evaluation for all materials: Chemfil Rock (*p* = 0.36), Equia (*p* = 0.13), Ketac Fil (*p* = 0.35), Ketac Molar Quick (*p* = 0.78), Riva LC (*p* = 0.054), Riva LC HV (*p* = 0.39), Riva SC Fast (*p* = 0.98), and Riva SC HV (*p* = 0.24). When between material results were considered at each time interval, the results found that at each time period that there were no significant difference between the materials identified.

## 4. Discussion

This evaluation involved the first long-term study of selected conventional and RMGI glass ionomer restorative products, including some materials not previously reported in the scientific literature. Thermal analysis techniques have been utilized to evaluate glass ionomer cement products [21,25,52,53,54,55] and this evaluation used a combined differential scanning calorimetry-thermogravimetric analysis technology (DSC/TGA) technology. Specimens were subjected to a 25–620 °C thermal challenge at a rate of 10 °C/min with simultaneous thermal and weight change data recorded. Assumptions made for this evaluation were that essentially the lower temperature enthalpy curves and weight change were associated with unbound water content. Additionally, it was also presumed that the higher temperature enthalpy and weight change were due to mainly the bound water species. Admittedly, these assumptions could be further refined with evolved gas analysis by either mass spectroscopy or infrared analysis, however these modalities were not available during this evaluation.

**Figure 2:**
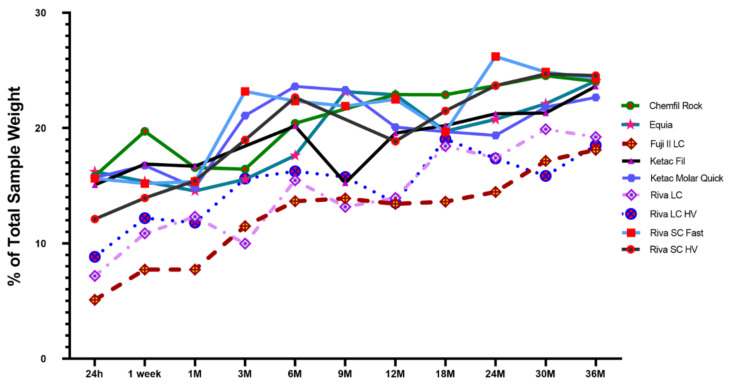
Mean Unbound Water Content (%). *n* = 12; Unbound water loss calculated as a percentage of total sample weight.

Graphical unbound water content over the evaluation is depicted in Figure 2.

The null hypothesis in regards to unbound water content was rejected, as some materials significantly increased unbound water content over the 36 months evaluation. Unbound water content was noted to stabilize and possibly annotate unbound water content capacity, but the content stability to that observed at 36 months was material specific. For example, similarity to that observed at 36 months was observed as soon as one week for Chemfil Rock, Riva LC and Riva LC HV; one month for Riva SC Fast, and three months with Fuji II LC, Ketac Molar Quick, and Riva SC HV. By six months none of the materials demonstrated a significant unbound water percentage content and were stable. Unbound water content increased with time and content stability was reached at approximately three months and six months for conventional GIC. However, loss of unbound water was still possible. The clinical implications of these results indicate that protective coatings are indicated to prevent unbound water loss.

**Figure 3:**
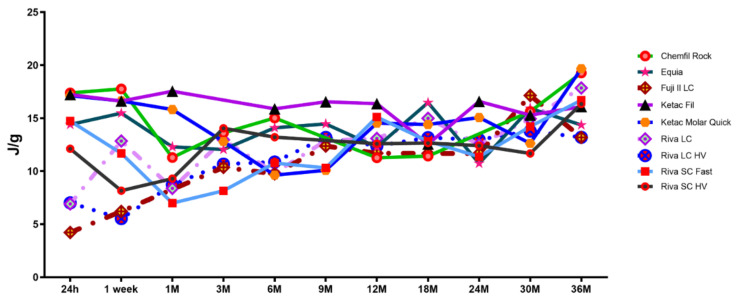
Mean Unbound Water Content Enthalpy (J/g); *n* = 12.

The mean enthalpy associated with the unbound water content is shown in Figure 3.

The null hypothesis was accepted for conventional GIC products but was rejected for the RMGI materials. No significant enthalpy increase to remove unbound water was noted with conventional GIC materials. This may reflect the loosely-held nature of unbound water and reinforces earlier findings of Nicholson and Czarnecka [37]. RMGI products were observed to initially require significantly less enthalpy for unbound water removal that increased to similarity with that required by conventional GIC materials by three months. This initial lower enthalpy requirement may be considered due to the early RMGI water content may be more associated as being cursorily retained, possibly similar to a recently-suggested, superficially bound water species [37]. Also, the water content mobile nature [47] may result in that internal water may be attracted and migrate to established hydrophilic regions, and thus require more energy to later remove [37]. The clinical implication of these results is that the unbound water species may be easily lost and that glass ionomer materials should be protected from this potential water loss as it may impair esthetic and/or mechanical properties.

**Figure 4:**
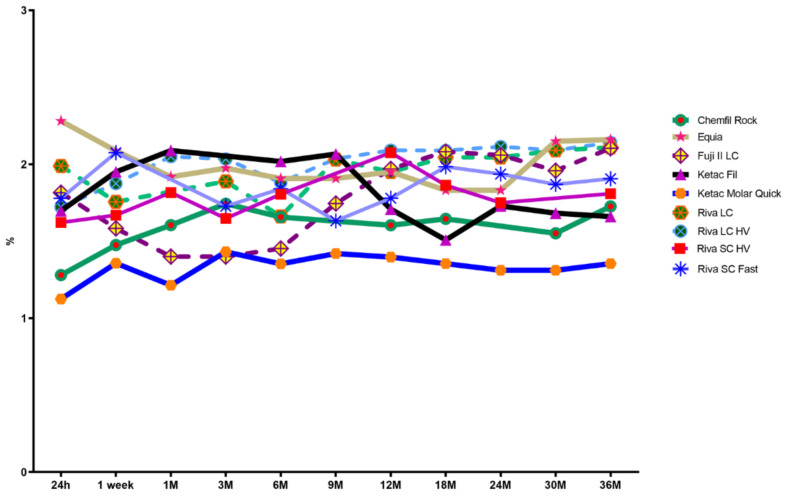
Mean Bound Water Content (%); *n* = 12; Bound water loss calculated as a percentage of total sample weight.

Water binding within glass ionomer materials has been described as a complex process [48] and assessment of bound water species content deserves additional consideration. When considered as a function of total sample weight, bound water content overall did not significantly increase for any material, with the collective maximum at 36 months noted as being approximately 2.1 percent or less of the total sample mass (Figure 4).

**Figure 5:**
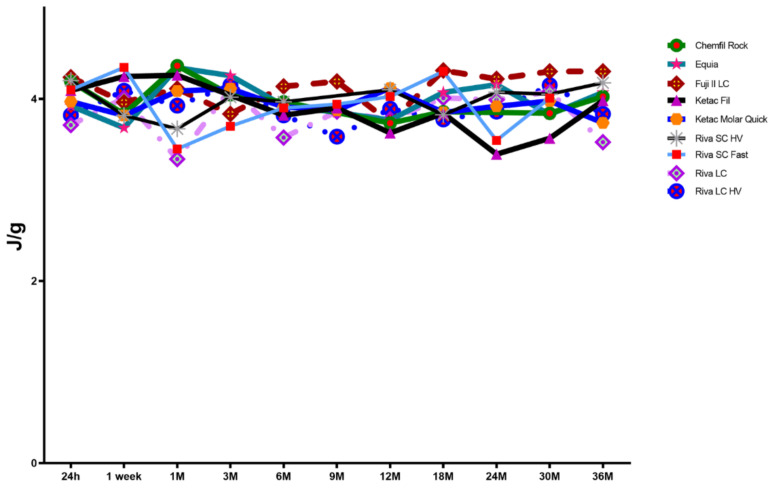
Mean Bound Water Loss Enthalpy (J/g); *n* = 12.

Likewise, bound water enthalpy was noted to exhibit very little change with and between materials as displayed in Figure 5, and the null hypothesis was accepted for bound water content and enthalpy.

However, bound water content results should also be considered within the limitations imposed with this study. Realistically, it should be considered that the higher temperature range associated with assessment of the bound water content under the conditions of this study may also include additional evolved components. Accordingly, unreacted liquid polyacrylic acid degradation temperature has been suggested to range from 158 °C [56], upwards to 255 °C [57], as well as 350 °C [58] and plausibly should not have affected the higher temperature range used for bound water content determination. However, changes associated with the glass transitions of the glass component are possible in the 424 to 500 °C thermal range [59], and polyacrylic polymer degradation has been identified to occur approximately at 425 °C [60]. Furthermore, aluminum and strontium complexed polyacrylic acid salts have also been reported to decompose between 425 and 520 °C [61,62,63]. When considering the RMGI materials, some methacrylate products have been reported to degrade starting at approximately 423 °C [64]. Understandably, bound water estimates in the concurrent presence of multiple thermal events can render assessments confusing, especially with weak thermal changes noted with some products which rendered delineation of individual transitions difficult.

The findings of this study contrast with previous reports. Wilson and Crisp estimated with early GIC products that the unbound water content to be in the range of that 18–28% of the total glass ionomer water content was loosely bound while tightly bound water was thought to be approximately 5% [45]. The observed unbound water content increase contradicts Berg et al. [65] who proposed that unbound water content decreases with time. The clinical implications of these results indicate that under the conditions of this study, unbound water may increase with time and that protective coatings are indicated to prevent this unbound water loss, especially during the first six months for conventional GIC products and three months for RMGI materials. Furthermore, this study reports that the bound water content does not increase with time, that contrasts what has been proposed to occur as the glass ionomer materials mature [46]. Also, bound water content less than the 5% suggested by Prosser and Wilson [66] but was similar to some of the values reported by Nicholson and Czarnecka [37]. Also, the phenomena of glass ionomer bound water increasing with time has been largely based from data observed from the very early GIC materials [46], not to mention silicate cements [67]. Accordingly, the early work by Wilson et al. [46] the glass ionomer materials demonstrated bound water proportion increases up to 24 h, but demonstrated little change afterwards. In this study little change in bound water content was found beyond 24 h, and it can also be conjectured that the newer materials used in this study were perhaps more reactive with all bound water migration completed by 24 h.

Limitations of this study include the fact that only a focused evaluation of GIC and RMGI materials was accomplished and the results of this evaluation may not be universally applied to all non-evaluated polyalkenoate materials. Furthermore, due to this study’s 36-month length some technique variation is admittedly to be expected. It should be realized that this study did not precisely follow clinical procedure, as the aim of this study was to possibly evaluate glass ionomer hydrogel matrix maturation via water content. To wit, material ample exposure to water was required and under the conditions of this study moisture availability was allowed by not placing protective coatings. To lessen chances of material loss due to solubility, specimens were not exposed to water during the early setting reaction. Furthermore, the specimens were then efficiently placed in a moisture rich PBS environment without full immersion. Admittedly, minor alteration of results is possible as this method did not control uniform moisture availability as well out rule out some weight change differences due to solubility. A further concern is that an varying oral environment could not be replicated. Dental restorations are constantly exposed to different textures and abrasion from food, as well as widely shifting pH values from the result of different beverages, not to mention lower pH levels involved with caries processes. The effect of these changes on the current data cannot be predicted at this time. However, the data in this study can hopefully establish a benchmark for future studies involving different oral environments. Some data was not available for five groups due to inadvertent loss of storage moisture. Fortunately, this loss of data was preceded and followed by ample data and could be produced by interpolation. Another limitation is that the initial weights of the freshly-prepared samples were not recorded as recording of this weight would require precise coordination of all weights to occur at this same time due to inherent water loss from the glass ionomer products after preparation. For the purpose of this study, all weight loss was assumed to be primarily due to water content loss, and loss was determined as a percentage of sample weight which provides some data normalization between samples. Furthermore, non-parametric data analysis was used in this evaluation due to concerns for the normal data distribution as well as variance inhomogeneity. However, non-parametric analysis will also compensate for inadvertent small sample size variation between groups. Definitive results for bound water is compromised due to possible overlapping reactions over the higher temperature range. Future studies should include means for deconvoluting and separation of these concurrent thermal processes. Also, some of the thermal results may have been below the detection thresholds of the technology used.

## 5. Conclusions

This study evaluated the water balance dynamics of six conventional and three resin modified glass ionomer restorative materials for up to 36 months. Under the conditions of this study:

(1) All materials demonstrated a significant increase in percentage of unbound water content over the evaluation period. The time that the unbound water content stabilized was material dependent, with the RMGI products reaching stability at three months and the conventional GIC products maintaining stability at six months. However, unbound water loss is still possible and protective coating placement and reevaluation is advised to maintain unbound water content.
(2) The conventional GIC products required no significant increase in enthalpy to remove the unbound water between 24 h and 36 months. Contrastingly, RMGI products demonstrated significant enthalpy increases required to remove unbound water.
(3) When considered as a percentage of total sample weight, there was no significant increase in bound water content as well as the enthalpy requirement to remove the bound water content past 24 h.

## Figures and Tables

**Table 1:** Products evaluated.

Product (Shade) Manufacturer	Type	Water/Powder Ratio	Powder	Liquid
Chemfil Rock (A2) Dentsply Sirona, York, PA, USA	GIC	0.442/0.12 (g/g) ^B^	Polycarboxylic acid * 10–25% Tartaric acid 0–2.5%	Butanedioic acid, 2-methylene-, polymer with 2-propenoic acid 10–25% Tartaric acid 3–10% (**)
Equia Fil (A2) GC America, Alsip, IL, USA	GIC	0.40/0.12 (g/g)	poly(acrylic acid) 2–5%	polybasic carboxylic acid* 5–10%
Fuji II LC (A2) GC America	RMGI	0.33/0.10 (g/g)	*	HEMA;* basic carboxylic acid* UDMA *
Ketac Fil Aplicap (A2) 3M Oral Care, St. Paul, MN, USA	GIC	3.6:1 ^C^	Glass Powder >99% *	Water 40–60% * Copolymer acrylic-maleic acid 30–50% * Tartaric acid 5–15% *
Ketac Molar Quick (A2) 3M Oral Care	GIC	3.4:1	Copolymer of acrylic-maleic acids 1–6% Dichlorodimethylsilane, reaction products with silica <2%	Water 60–65%% * Copolymer acrylic-maleic acid 30–40% * Tartaric acid 5–10% *
Riva LC (A2) SDI North America, Itasca, IL, USA.	RMGI	0.42/0.14 (g/g)	glass powder * 95–100%	HEMA 20–30% acrylic acid homopolymer 15–25% dimethacrylate cross-linker 10–25% acidic monomer 10–20% tartaric acid 1–5%
Riva LC HV (A2) SDI North America	RMGI	0.43/0.13 (g/g)	glass powder * 93–100%	HEMA 15–25% acrylic acid homopolymer 15–25% dimethacrylate cross-linker 10–25% acidic monomer 10–20% tartaric acid 1–5%
Riva SC Fast (A2) SDI North America	GIC	0.42/0.13 (g/g)	fluoro aluminosilicate glass 90–95%	acrylic acid homopolymer 20–30% tartaric acid 10–15%
Riva SC HV (A2) SDI North America	GIC	0.50/0.13 (g/g)	fluoro aluminosilicate glass 90–95% acrylic acid homopolymer 5–10%	acrylic acid homopolymer 20–30% tartaric acid 10–15%

GIC = Conventional (e.g., water based—no resin components) glass ionomer (Polyalkenoate) cement restorative material; RMGI = Resin modified glass ionomer; HEMA = 2-hydroxyethyl methacrylate; UDMA = Urethane dimethacrylate; * annotates trade secret; w/p ratio and product contents obtained from manufacturer supplied information and Safety Data Sheets; liquid balance assumed to be water (**) = Safety Data Sheets not consistent internationally.

**Table 2:** Mean unbound water content (%).

	24 h	1 Week	1 Month	3 Months	6 Months	9 Months	12 Months	18 Months	24 Months	30 Months	36 Months
**Chemfil Rock**	15.8 (0.4) A a	19.7 (1.1) A ab	16.6 (1.7) A a	16.4 (2.0) AB a	20.4 (2.7) AB ab	*	22.9 (2.5) A b	22.9 (1.7) A b	*	24.5 (2.4) A b	24.1 (2.3) A b
**Equia**	16.2 (2.3) A bc	15.4 (2.2) ABC bc	14.6 (1.4) A c	15.6 (1.9) AB bc	17.6 (2.1) ABC abc	23.2 (5.2) A a	22.9 (3.4) A a	19.7 (5.2) AB ab	20.8 (4.4) AB ab	22.1 (6.5) AB a	24.1 (5.0) A a
**Fuji II LC**	5.1 (0.8) C c	7.7 (0.5) D bc	7.9 (0.6) B bc	11.5 (2.8) B abc	13.7 (2.4) C ab	13.9 (3.1) B ab	13.4 (3.1) C ab	13.6 (2.9) AB ab	14.5 (2.5) C ab	17.2 (4.1) B a	18.1 (3.9) B a
**Ketac Fil**	15.1 (4.0) AB c	16.9 (1.4) AB bc	16.7 (3.5) A bc	*	20.2 (3.5) AB ab	15.2 (1.9) B c	19.6 (2.3) AB ab	20.2 (3.7) AB abc	21.2 (2.0) AB ab	21.3 (3.0) AB ab	23.6 (4.2) AB a
**Ketac Molar Quick**	15.7 (1.4) A bc	16.8 (1.8) AB bc	14.9 (1.8) A c	21.1 (4.0) A a	23.6 (5.1) A a	23.3 (2.7) A a	20.1 (2.0) AB ab	19.7 (2.8) AB ab	19.4 (2.0) ABC bc	21.8 (2.6) AB a	22.7 (3.7) AB a
**Riva LC**	6.9 (1.9) C c	12.8 (4.4) CD abc	8.4 (2.2) AB abc	13.0 (2.3) B abc	9.8 (3.0) BC bc	12.9 (5.3) B abc	13.1 (3.8) B abc	15.0 (4.5) BC ab	12.5 (2.2) BC abc	14.9 (3.3) AB ab	17.9 (2.9) B a
**Riva LC HV**	8.8 (1.3) BC b	12.2 (3.3) BCD b	11.8 (1.7) AB ab	15.6 (3.7) AB a	16.3 (2.4) BC a	15.8 (4.4) B a	13.5 (1.7) B ab	19.0 (5.4) ABC a	17.4 (1.2) BC a	15.9 (1.9) B a	18.5 (2.1) B a
**Riva SC Fast**	15.6 (1.1) A bc	15.2 (1.1) BC c	19.5 (3.3) A abc	23.2 (4.1) A a	22.4 (5.2) A ab	21.9 (4.2) A abc	22.5 (3.2) A a	19.7 (2.0) AB abc	26.2 (4.6) A a	24.9 (3.0) A a	24.3 (2.6) A a
**Riva SC HV**	12.1 (0.5) AB c	13.9 (2.2) BCD c	15.4 (2.0) A bc	19.0 (2.4) A abc	22.7 (3.9) A ab	*	18.9 (1.8) AB bc	21.5 (2.8) A ab	23.7 (2.9) A a	24.7 (2.4) A a	24.6 (2.1) A a

*n* = 12; * = no data available; Unbound water loss calculated as a percentage of total sample weight. Capital letters identify similar groups per column while lower case letters identify similar groups per row (Kruskal-Wallis/Dunn’s; *p* = 0.05).

**Table 3:** Mean Unbound Water Enthalpy (J/g).

	24 h	1 Week	1 Month	3 Months	6 Months	9 Months	12 Months	18 Months	24 Months	30 Months	36 Months
**Chemfil Rock**	17.4 (2.3) A ab	17.8 (4.7) A ab	11.3 (2.0) AB c	13.6 (3.1) A bc	15.0 (3.1) A abc	*	11.3 (3.4) A c	11.4 (3.2) A c	*	15.6 (2.1) A abc	19.3 (2.1) A a
**Equia**	14.4 (3.4) A a	15.5 (3.0) A a	12.3 (1.7) AB a	12.0 (2.5) AB a	14.1 (2.4) AB a	14.5 (3.7) AB a	12.3 (2.9) A a	16.5 (3.3) A a	10.7 (3.5) B a	15.9 (3.2) A a	14.4 (2.3) B a
**Fuji II LC**	4.2 (1.5) B c	6.2 (1.7) B c	*	10.4 (2.0) AB bc	9.7 (2.5) B bc	12.4 (2.5) AB ab	11.7 (3.6) A ab	11.7 (3.2) A ab	11.7 (2.5) AB ab	17.1 (2.8) A a	13.2 (2.7) B ab
**Ketac Fil**	17.2 (2.5) A a	16.6 (3.0) A a	17.5 (5.0) A a	*	15.9 (4.1) A a	16.5 (3.6) A a	16.4 (3.7) A a	12.6 (3.9) A a	16.6 (3.1) A a	15.3 (4.5) A a	16.1 (2.3) AB a
**Ketac Molar Quick**	17.1 (4.2) A a	16.6 (3.4) A a	15.8 (4.2) A ab	12.8 (3.2) A ab	9.6 (1.7) B b	10.1 (2.3) B b	14.5 (5.6) A ab	14.4 (4.2) A ab	15.1 (6.1) AB ab	12.6 (5.1) A ab	19.7 (3.8) A a
**Riva LC**	6.9 (1.9) B b	12.8 (4.4) AB ab	8.4 (2.2) B ab	13.0 (2.3) AB ab	9.8 (3.0) AB ab	13.0 (5.3) B ab	13.1 (3/8) A ab	15.0,(4.5) A a	12.5 (2.2) AB ab	15.0 (3.4) A a	18.0 (3.0) AB a
**Riva LC HV**	7.0 (2.5) B b	5.5 (1.5) B b	8.7 (1.8) B ab	10.7 (3.0) AB ab	10.9 (3.8) AB ab	13.2 (3.0) AB a	12.4 (3.4) A a	13.2 (3.4) A a	13.0 (3.5) AB a	13.8 (2.1) A a	13.2 (3.0) B a
**Riva SC Fast**	14.7 (2.6) A a	11.7 (2.3) AB ab	7.0 (2.0) B b	8.2 (1.8) B b	10.8 (3.0) AB ab	10.3 (2.9) B ab	15.1 (3.5) A a	12.8 (4.1) A ab	11.4 (3.3) B ab	14.2 (1.9) A a	16.7 (3.4)AB a
**Riva SC HV**	12.1 (1.8) AB ab	8.20 (2.5) A b	9.30 (2.1) B b	14.0 (5.0) A ab	13.2 (3.5) AB ab	*	12.6 (4.8) A ab	12.7 (4.0) A ab	12.4 (3.6) AB ab	11.7 (4.2) A ab	16.3 (4.2) AB a

*n* = 12; * = no data available; Capital letters identify similar groups per column, lower case letters identify similar groups per row (Kruskal-Wallis/Dunn’s, *p* = 0.05).

**Table 4:** Mean Bound Water Loss (%).

	24 h	1 Week	1 Month	3 Months	6 Months	9 Months	12 Months	18 Months	24 Months	30 Months	36 Months
**Chemfil Rock**	1.3 (0.2) B a	1.5 (0.4) B a	1.6 (0.2) AB a	1.7 (0.4) AB a	1.6 (0.2) AB a	*	1.6 (0.3) B a	1.6 (0.2) AB a	*	1.5 (0.6) B a	1.7 (0.4) B a
**Equia**	2.3 (0.5) A a	2.1 (0.3) A a	1.9 (0.2) A a	2.0 (0.1) A a	1.9 (0.1) A	1.9 (0.2) AB a	1.9 (0.2) A a	1.8 (0.1) AB a	1.8 (0.1) AB a	2.1 (0.2) A a	2.1 (0.2) A a
**Fuji II LC**	1.8 (0.3) AB ab	1.6 (0.1) AB ab	1.4 (0.2) B b	1.4 (0.1) B b	1.4 (0.1) B b	1.7 (0.3) AB ab	2.0 (0.1) A a	2.1 (0.2) A a	2.1 (0.1) A a	1.9 (0.4) AB a	2.1 (0.3) A a
**Ketac Fil**	1.7 (0.3) AB ab	1.9 (0.3) AB a	2.1 (0.3) A a	*	2.0 (0.3) A a	2.1 (0.1) A a	1.7 (0.3) AB ab	1.5 (0.3) AB b	1.7 (0.2) AB ab	1.7 (0.2) AB ab	1.6 (0.2) B ab
**Ketac Molar Quick**	1.1 (0.1) B a	1.3 (0.2) B a	1.2 (0.1) B a	1.4 (0.4) B a	1.3 (0.2) B a	1.4 (0.2) B a	1.4 (0.1) B a	1.3 (0.3) B a	1.3 (0.1) B a	1.3 (0.1) B a	1.3 (0.1) B a
**Riva LC**	2.0 (0.2) AB a	1.7 (0.3) AB a	*	1.9 (0.3) AB a	1.6 (0.3) AB a	2.0 (0.2) A a	1.9 (0.1) A a	2.0 (0.3) A a	2.0 (0.2) A a	2.1 (0.2) A a	2.1 (0.1) A a
**Riva LC HV**	1.7 (0.3) AB a	1.9 (0.3) AB a	2.0 (0.2) A a	2.0 (0.1) A a	1.9 (0.2) A a	2.0 (0.1) A a	2.1 (0.1) A a	2.1 (0.2) A a	2.1 (0.1) A a	2.1 (0.2) A a	2.1 (0.4) A a
**Riva SC Fast**	1.8 (0.3) AB a	2.1 (0.3) A a	*	1.7 (0.3) AB a	1.8 (0.2) AB a	1.6 (0.2) AB a	1.8 (0.4) AB a	2.0 (0.2) A a	1.9 (0.3) AB a	1.8 (0.3) AB a	1.9 (0.1) A a
**Riva SC HV**	1.6 (0.4) AB a	1.6 (0.1) AB a	1.8 (0.3) AB a	1.6 (0.3) AB a	1.8 (0.2) AB a	*	2.1 (0.2) A a	1.8 (0.1) AB a	1.7 (0.3) AB a	*	1.8 (0.2) AB a

*n* = 12; Bound water loss calculated as a percentage of total sample weight; * = no data available; Capital letters identify similar groups per column, lower case letters identify similar groups per row (Kruskal-Wallis/Dunn’s, *p* = 0.05).

**Table 5:** Mean Bound Water Enthalpy (J/g).

	24 h	1 Week	1 Month	3 Months	6 Months	9 Months	12 Months	18 Months	24 Months	30 Months	36 Months
**Chemfil Rock**	4.19 (1.1) A a	3.83 (0.84) A a	4.36 (1.2) A a	4.0 (1.1) A a	4.0 (0.9) A a	*	3.73 (0.9) A a	3.85 (0.7) A a	*	3.84 (1.1) A a	4.02 (0.7) A a
**Equia**	3.92 (0.4) A a	3.68 (0.8) A a	4.34 (0.5) A a	4.25 (1.8) A a	3.92 (0.7) A a	3.85 (1.3) A a	3.76 (0.5) A a	4.07 (0.8) A a	4.15 (0.6) A a	3.82 (1.1) a	4.07 (0.8) A a
**Fuji II LC**	4.23 (1.2) A a	4.0 (0.8) A a	*	3.83 (1.4) A a	4.1 (1.0) A a	4.20 (0.6) A a	3.74 (1.2) A a	4.31 (1.0) A a	4.2 (1.4) A a	4.30 (1.5) A a	4.31 (1.0) A a
**Ketac Fil**	4.10 (0.9) A a	4.24 (1.2) A a	4.26 (2.1) A a	*	3.82 (0.5) A a	3.90 (1.1) A a	3.62 (1.1) A a	3.84 (0.8) A a	3.40 (0.5) A a	3.56 (0.4) A a	3.98 (0.9) A a
**Ketac Molar Quick**	3.97 (0.5) A a	3.81 (0.4) A a	4.1 (0.5) A a	4.1 (1.3) A a	3.91 (1.0) A a	3.87 (0.6) A a	4.12 (1.4) A a	3.85 (1.1) A a	3.91 (0.5) A a	4.0 (0.8) A a	3.73 (2.1) A a
**Riva LC**	3.71 (0.8) A a	4.01 (1.1) A a	3.33 (1.3) A a	4.11 (1.3) A a	3.57 (1.3) A a	3.86 (1.0) A a	3.77 (1.1) A a	4.00 (0.9) A a	4.1 (1.4) A a	4.07 (1.3) A a	3.52 (0.9) A a
**Riva LC HV**	3.82 (1.4) A a	4.08 (1.5) A a	3.92 (0.8) A a	4.15 (0.2) A a	3.81 (0.7) A a	3.58 (0.5) A a	3.90 (0.9) A a	3.77 (1.2) A a	3.86 (1.0) A a	4.15 (1.0) A a	3.83 (0.9) A a
**Riva SC Fast**	4.10 (0.8) A a	4.34 (0.4) A a	3.45 (0.5) A a	3.70 (0.6) A a	3.9 (1.5) A a	3.94 (1.4) A a	4.02 (1.2) A a	4.30 (1.5) A a	3.54 (1.2) A a	4.00 (1.4) A a	4.10 (0.8) A a
**Riva SC HV**	4.20 (1.1) A a	3.82 (0.7) A a	3.67 (0.9) A a	4.01 (1.1) A a	4.00 (0.6) A a	*	4.10 (0.5) A a	3.82 (0.6) A a	4.07 (0.9) A a	4.05 (0.9) A a	4.17 (1.0) A a

*n* = 12; * = no data available; Capital letters identify similar groups per column, lower case letters identify similar groups per row (Kruskal-Wallis/Dunn’s, *p* = 0.05).

## Data Availability

The data presented in this study are available on request from the corresponding author.

## References

[1] Da Mata C., McKenna G., Anweigi L., Hayes M., Cronin M., Woods N., O’Mahony D., Allen P.F. (2019). An RCT of atraumatic restorative treatment for older adults: 5 year results. J. Dent..

[2] Frencken J.E., Leal S.C., Navarro M.F. (2012). Twenty-five-year atraumatic restorative treatment (ART) approach: A comprehensive overview. Clin. Oral. Investig..

[3] De Amorim R.G., Frencken J.E., Raggio D.P., Chen X., Hu X., Leal S.C. (2018). Survival percentages of atraumatic restorative treatment (ART) restorations and sealants in posterior teeth: An updated systematic review and meta-analysis. Clin. Oral. Investig..

[4] Heck K., Frasheri I., Diegritz C., Manhart J., Hickel R., Fotiadou C. (2020). Six-year results of a randomized controlled clinical trial of two glass ionomer cements in class II cavities. J. Dent..

[5] Miletić I., Baraba A., Basso M., Pulcini M.G., Marković D., Perić T., Ozkaya C.A., Turkun L.S. (2020). Clinical Performance of a Glass-Hybrid System Compared with a Resin Composite in the Posterior Region: Results of a 2-year Multicenter Study. J. Adhes. Dent..

[6] Gurgan S., Kutuk Z.B., Yalcin Cakir F., Ergin E. (2020). A randomized controlled 10 years follow up of a glass ionomer restorative material in class I and class II cavities. J. Dent..

[7] Wilson A.D., Kent B.E. (1971). The glass-ionomer cement, a new translucent dental filling material. J. Appl. Chem..

[8] Wilson A.D., Batchelor R.F. (1967). Dental silicate cements I. The chemistry of erosion. J. Dent. Res..

[9] Nicholson J.W. (1998). Chemistry of glass-ionomer cements: A review. Biomaterials.

[10] Baig M.S., Fleming G.J.P. (2015). Conventional glass-ionomer materials: A review of the developments in glass powder, polyacid liquid and the strategies of reinforcement. J. Dent..

[11] Khoroushi M., Keshani F. (2013). A review of glass-ionomers: From conventional glass-ionomer to bioactive glass-ionomer. Dent. Res. J..

[12] Nicholson J.W., Sidhu S.K., Czarnecka B. (2020). Enhancing the Mechanical Properties of Glass-Ionomer Dental Cements: A Review. Materials.

[13] Najeeb S., Khurshid Z., Zafar M.S., Khan A.S., Zohaib S., Martí J.M., Sauro S., Matinlinna J.P., Rehman I.U. (2016). Modifications in Glass Ionomer Cements: Nano-Sized Fillers and Bioactive Nanoceramics. Int. J. Mol. Sci..

[14] Tüzüner T., Dimkov A., Nicholson J.W. (2019). The effect of antimicrobial additives on the properties of dental glass-ionomer cements: A review. Acta. Biomater. Odontol. Scand..

[15] Kantovitz K.R., Fernandes F.P., Feitosa I.V., Lazzarini M.O., Denucci G.C., Gomes O.P., Giovani P.A., Moreira K.M.S., Pecorari V.G.A., Borges A.F.S. (2020). TiO_2_ nanotubes improve physico-mechanical properties of glass ionomer cement. Dent. Mater..

[16] Hatton P.V., Brook I.M. (1992). Characterization of the ultrastructure of glass-ionomer (poly-alkenoate) cement. Br. Dent. J..

[17] Cattani-Lovente M.A., Godin C., Meyer J.M. (1994). Mechanical behavior of glass ionomer cements affected by the long-term storage in water. Dent. Mater..

[18] Matsuya S., Maeda T., Ohta M. (1996). IR and NMR analyses of hardening and maturation of glass-ionomer cement. J. Dent. Res..

[19] Mitra S.B. (1991). Adhesion to dentin and physical properties of a light-cured glass-ionomer liner/base. J. Dent. Res..

[20] Friedl K.H., Powers J.M., Hiller K.A. (1995). Influence of different factors on bond strength of hybrid ionomers. Oper. Dent..

[21] Agha A., Parker S., Patel M. (2020). Polymerization shrinkage kinetics and degree of conversion of commercial and experimental resin modified glass ionomer luting cements (RMGICs). Dent. Mater..

[22] Anstice H.M., Nicholson J.W. (1994). Studies in the setting of polyelectrolyte materials—Part II: The effect of organic compounds on a glass poly(alkenoate) cement. J. Mater. Sci. Mater. Med..

[23] Wan A.C., Yap A.U., Hastings G.W. (1999). Acid-base complex reactions in resin-modified and conventional glass ionomer cements. J. Biomed. Mater. Res..

[24] Kakaboura A., Eliades G., Palaghias G. (1996). An FTIR study on the setting mechanism of resin-modified glass ionomer restoratives. Dent. Mater..

[25] Berzins D.W., Abey S., Costache M.C., Wilkie C.A., Roberts H.W. (2010). Resin-modified glass-ionomer setting reaction competition. J. Dent. Res..

[26] Roberts H.W., Berzins D.C. (2015). Early reaction kinetics of contemporary glass-ionomer restorative materials. J. Adhes. Dent..

[27] Yelamanchili Y., Darvell B.W. (2008). Network competition in a resin-modified glass-ionomer cement. Dent. Mater..

[28] Kim Y.K., Kim K.H., Kwon T.Y. (2015). Setting Reaction of Dental Resin-Modified Glass Ionomer Restoratives as a Function of Curing Depth and Post irradiation Time. J. Spectrosc..

[29] Young A., Sherpa G., Pearson B., Schottlander Waters D.N. (2000). Use of Raman Spectroscopy in the Characterisation of the Acid–Base Reaction in Glass-Ionomer Cements. Biomaterials.

[30] Stamboulis A., Matsuya S., Hill R.G., Law R.V., Udoh K., Nakagawa M. (2006). MAS-NMR spectroscopy studies in the setting reaction of glass ionomer cements. J. Dent..

[31] Šantić A., Čalogović A.M., Pavić L., Gladić J., Vučić Z., Lovrić D., Prskalo K., Janković B., Tarle Z., Moguš-Milanković A. (2015). New Insights into the Setting Processes of Glass Ionomer Cements from Analysis of Dielectric Properties. J. Am. Ceram. Soc..

[32] Wren A.W., Kidari A., Cummins N.M., Towler M.R. (2010). A spectroscopic investigation into the setting and mechanical properties of titanium containing glass polyalkenoate cements. J. Mater. Sci. Mater. Med..

[33] Zainuddin N., Karpukhina N., Hill R.G., Law R.V. (2009). A long-term study on the setting reaction of glass ionomer cements by (27)Al MAS-NMR spectroscopy. Dent. Mater..

[34] Berg M.C., Jacobsen J., Momsen N.C.R., Benetti A.R., Telling M.T.F., Seydel T., Bordallo H.N. (2016). Water dynamics in glass ionomer cements. Eur. Phys. J. Spec. Top..

[35] Akashi A., Matsuya Y., Unemori M., Akamine A. (1999). The relationship between water absorption characteristics and the mechanical strength of resin-modified glass-ionomer cements in long-term water storage. Biomaterials.

[36] Nicholson J.W. (2018). Maturation processes in glass-ionomer dental cements. Acta Biomater. Odont. Scand..

[37] Nicholson J.W., Czarnecka B. (2008). Kinetic studies of water uptake and loss in glass-ionomer cements. J. Mater. Sci. Mater. Med..

[38] Feilzer A.J., Kakaboura A.I., de Gee A.J., Davidson C.L. (1995). The influence of water sorption on the development of setting shrinkage stress in traditional and resin-modified glass ionomer cements. Dent. Mater..

[39] Hotta M., Hirukawa H., Yamamoto K. (1992). Effect of coating materials on restorative glass-ionomer cement surface. Oper. Dent..

[40] Jevnikar P., Sersâ I., Sepe A., Jarh O., Funduk N. (2000). Effect of surface coating on water migration into resin-modified glass ionomer cements: A magnetic resonance micro-imaging study. Magn. Reason. Med..

[41] Miyazaki M., Moore B.K., Onose H. (1996). Effect of surface coatings on flexural properties of glass ionomers. Eur. J. Oral. Sci..

[42] Aydın N., Karaoğlanoğlu S., Aybala-Oktay E., Çetinkaya S., Erdem O. (2020). Investigation of water sorption and aluminum releases from high viscosity and resin modified glass ionomer. J. Clin. Exp. Dent..

[43] Gorseta K., Glavina D., Skrinjaric T., Czarnecka B., Nicholson J.W. (2016). The effect of petroleum jelly, light-cured varnish and different storage media on the flexural strength of glass ionomer dental cements. Acta Biomater. Odontol. Scand..

[44] Hornsby P.R. (1980). Dimensional stability of glass-ionomer cements. J. Chem. Technol. Biotechnol..

[45] Wilson A.D., Crisp S. (1975). Ionomer Cements. Br. Polym. J..

[46] Wilson A.D., Crisp S., Paddon J.M. (1981). Hydration of a glass ionomer (ASPA) cement. Brit. Polym. J..

[47] Van Meerbeck B., Yoshida Y., Inoue S., Munck J., Landuyt K., Lambrechts P. (2006). Glass ionomer adhesion: The mechanisms at the interface. J. Dent..

[48] Czarnecka B., Klos J., Nicholson J.W. (2015). The effect of ionic solutions on the uptake and water-binding behaviour of glass-ionomer dental cements. Ceram. Silik..

[49] Faroud M.A., Stamboulis A. (2014). Nanoclay addition to conventional glass-ionomer cements: Influence on properties. Eur. Dent. J..

[50] Tadjiev D., Hand R. (2010). Surface hydration and nanoindentation of silicate glasses. J. Non-Cryst. Solids.

[51] ISO (2017). 9917-2: Water Based Cements—Part 2: Resin-Modified Cements.

[52] Pagano S., Chieruzzi M., Balloni S., Lombardo G., Torre L., Bodo M., Cianetti S., Marinucci L. (2019). Biological, thermal and mechanical characterization of modified glass ionomer cements: The role of nanohydroxyapatite, ciprofloxacin and zinc l-carnosine. Mater. Sci. Eng. C. Mater. Biol. Appl..

[53] Bourke A.M., Walls A.W., McCabe J.F. (1992). Light-activated glass polyalkenoate (ionomer) cements: The setting reaction. J. Dent..

[54] Kilpatrick N.M., McCabe J.F., Murray J.J. (1994). Factors that influence the setting characteristics of encapsulated glass ionomer cements. J. Dent..

[55] Micelli F., Maffezzoli A., Terzi R., Luprano V.A. (2001). Characterization of the kinetic behavior of resin modified glass-ionomer cements by DSC, TMA and ultrasonic wave propagation. J. Mater. Sci. Mater. Med..

[56] Khalil S.K., Atkins E.D. (1998). Investigation of glass-ionomer cements using differential scanning calorimetry. J. Mater. Sci. Mater. Med..

[57] Zelmer C., Wang D.K., Keen I., Hill D.J., Symons A.L., Walsh L.J., Rasoul F. (2016). Synthesis and characterization of POSS-(PAA)8 star copolymers and GICs for dental applications. Dent. Mater..

[58] McNeill I.C., Sadeghi S.M.T. (1990). Thermal stability and degradation mechanisms of poly(acrylic acid) and its salts: Part 1—Poly(-acrylic acid). Polym. Degrad. Stab..

[59] Pedersen M.T., Tian K.V., Dobó-Nagy C., Chass G.A., Greaves G.N., Yue Y. (2015). Phase separation in an ionomer glass: Insight from calorimetry and phase transitions. J. Non-Cryst. Solids.

[60] Caárdenas G., Munñoz C., Carbacho H. (2000). Thermal Properties and TGA−FTIR Studies of Polyacrylic and Polymethacrylic Acid Doped with Metal Clusters. Eur. Polym. J..

[61] Roberts H., Berzins D. (2013). Thermal Analysis of Contemporary Glass-Ionomer Restorative Materials. J. Therm. Anal. Calorim..

[62] McNeil I.C., Sadeghi S.M.T. (1990). Thermal stability and degradation mechanisms of poly(acrylic acid) and its salts: Part 3—Magnesium and calcium salts. Polym. Degrad. Stab..

[63] Nicholson J.W., Wilson A.D. (1987). Thermal behaviour of films of partially neutralized poly(acrylic acid). 1: Influence of metal ions. Br. Polym. J..

[64] Ferrante M., Petrini M., Trentini P., Ciavarelli L., Spoto G. (2010). Thermal analysis of light curing composites. J. Therm. Anal. Calorim..

[65] Berg M.C., Benetti A.R., Telling M.T.F., Seydel T., Yu D., Daemen L.L., Bordallo H.N. (2018). Nanoscale Mobility of Aqueous Polyacrylic Acid in Dental Restorative Cements. ACS Appl. Mater. Interfaces.

[66] Prosser H.J., Wilson A.D. (1979). Litho-ionomer cements and their technological applications. J. Chem. Techol. Biotechnol..

[67] Wilson A.D., Kent B.E., Batchelor R.F., Scott B.G., Lewis B.G. (1970). Dental silicate cements. XII. The role of water. J. Dent. Res..

